# A Serious Game for the Assessment of Visuomotor Adaptation Capabilities during Locomotion Tasks Employing an Embodied Avatar in Virtual Reality

**DOI:** 10.3390/s23115017

**Published:** 2023-05-24

**Authors:** Vladimiro Suglia, Antonio Brunetti, Guido Pasquini, Mariapia Caputo, Tommaso Maria Marvulli, Elena Sibilano, Sara Della Bella, Paola Carrozza, Chiara Beni, David Naso, Vito Monaco, Giovanna Cristella, Vitoantonio Bevilacqua, Domenico Buongiorno

**Affiliations:** 1 Department of Electrical and Information Engineering (DEI), Polytechnic University of Bari, 70126 Bari, Italy; vladimiro.suglia@poliba.it (V.S.); antonio.brunetti@poliba.it (A.B.); m.caputo12@studenti.poliba.it (M.C.); t.marvulli@studenti.poliba.it (T.M.M.); elena.sibilano@poliba.it (E.S.); david.naso@poliba.it (D.N.); domenico.buongiorno@poliba.it (D.B.); 2 Apulian Bioengineering s.r.l., 70026 Modugno, Italy; 3 IRCCS Fondazione Don Carlo Gnocchi ONLUS, 50143 Florence, Italy; gpasquini@dongnocchi.it (G.P.); sdellabella@dongnocchi.it (S.D.B.); pcarrozza@dongnocchi.it (P.C.); cbeni@dongnocchi.it (C.B.); 4 The Biorobotics Institute, Department of Excellence in Robotics and AI, Scuola Superiore Sant’Anna, 56127 Pisa, Italy; vito.monaco@santannapisa.it

**Keywords:** serious game, visuomotor adaptation, avatar, virtual reality, body tracking, Azure Kinect, gait, motion capture, visual feedback, human skeleton

## Abstract

The study of visuomotor adaptation (VMA) capabilities has been encompassed in various experimental protocols aimed at investigating human motor control strategies and/or cognitive functions. VMA-oriented frameworks can have clinical applications, primarily in the investigation and assessment of neuromotor impairments caused by conditions such as Parkinson’s disease or post-stroke, which affect the lives of tens of thousands of people worldwide. Therefore, they can enhance the understanding of the specific mechanisms of such neuromotor disorders, thus being a potential biomarker for recovery, with the aim of being integrated with conventional rehabilitative programs. Virtual Reality (VR) can be entailed in a framework targeting VMA since it allows the development of visual perturbations in a more customizable and realistic way. Moreover, as has been demonstrated in previous works, a serious game (SG) can further increase engagement thanks to the use of full-body embodied avatars. Most studies implementing VMA frameworks have focused on upper limb tasks and have utilized a cursor as visual feedback for the user. Hence, there is a paucity in the literature about VMA-oriented frameworks targeting locomotion tasks. In this article, the authors present the design, development, and testing of an SG-based framework that addresses VMA in a locomotion activity by controlling a full-body moving avatar in a custom VR environment. This workflow includes a set of metrics to quantitatively assess the participants’ performance. Thirteen healthy children were recruited to evaluate the framework. Several quantitative comparisons and analyses were run to validate the different types of introduced visuomotor perturbations and to evaluate the ability of the proposed metrics to describe the difficulty caused by such perturbations. During the experimental sessions, it emerged that the system is safe, easy to use, and practical in a clinical setting. Despite the limited sample size, which represents the main limitation of the study and can be compensated for with future recruitment, the authors claim the potential of this framework as a useful instrument for quantitatively assessing either motor or cognitive impairments. The proposed feature-based approach gives several objective parameters as additional biomarkers that can integrate the conventional clinical scores. Future studies might investigate the relation between the proposed biomarkers and the clinical scores for specific disorders such as Parkinson’s disease and cerebral palsy.

## 1. Introduction

Visuomotor adaptation (VMA) is a form of sensorimotor learning that allows humans to learn how to adapt to, or correct for, an external visual perturbation [1]. Learning how to compensate for the effects of external perturbations relies on the formation of an internal model that computes the difference between anticipated errors of the intended movement and actual errors from sensory feedback, which is then used to plan the next motor action.

A number of studies have proposed different experimental protocols with the aim of evaluating visuomotor adaptation abilities for a large variety of tasks, such as drawing [2,3,4], target reaching [5,6], and walking [7,8,9]. Since VMA mainly deals with the learning of the motor adjustment to be executed for reducing the visual perturbation effects to accomplish the given task [1], visual perturbations eliciting VMA may be included in experimental protocols to investigate motor learning and motor control abilities of healthy subjects [10,11,12,13,14,15,16,17]. Moreover, several studies have investigated how to integrate VMA evaluation into cognitive assessment protocols. In fact, visuomotor adaptation has been found to be correlated with cognitive functions, e.g., working memory, executive functions, and processing speed [18,19,20,21,22,23,24], and is also related to motion awareness and cognitive load [25,26].

Experimental setups for VMA capability evaluation have also been largely employed in clinical scenarios for multiple purposes, such as to evaluate the effects of aging on motor skills—e.g., human balance, gait parameters, or adaptive processes [7,27,28]—and to verify the relationship between the decline in adaptation and age-related deterioration of cognitive functions [29,30,31,32]. Experiments entailing VMA have also been proposed to investigate and assess the capabilities of subjects affected by neuromotor disorders, such as: people with cerebellar degeneration [33], children with autism spectrum and developmental coordination disorder [34,35], teenagers with cerebral palsy [36], patients affected by Parkinson’s disease [37,38,39], hemispheric lateralization of stroke survivors [40], multiple sclerosis patients [41], dystonic subjects [42], autistic individuals [43], and people affected by essential tremors [44].

A typical VMA experimental protocol encompasses a phase in which the subject under evaluation is asked to perform a motor task in a scenario featuring an altered condition. A framework targeting VMA is mainly composed of three fundamental elements: the scenario, or the environment, in which the goal-oriented task is executed; the motor command input system, which is necessary to detect the performed motor action in the real world and encode it in an action within the scenario/environment; and a set of possible perturbations used to alter the “normal” mapping between the real world and the scenario/environment. These aspects have to be tailored to the research purpose as well as to the human body part that is involved during the requested motor task. VMA may be evaluated in a real scenario by interposing prism lenses between the subject’s view and the workspace [45]; on the other hand, the motor task can be visualized in a virtual environment (VE), which can be provided to the user by means of either a screen [5,6,7,8,9,15,19,22,28,38,40,42,46], which is optionally combined with a mirror projecting computer-generated images on the workspace [5,6,15,19,22,28,38,40,42], or a head-mounted display (HMD) [17,27,47,48,49,50]. Hence, while the visual feedback given to the user is altered, the activity to execute might consist of reaching a target through a cursor [5,6,15,19,22,38,40,42,51], or in keeping balance [7,8,9,27,47,48]. Moreover, the motor actions performed by the subject can be guided in different ways, such as: by recording the position of a device mediating the interaction, e.g., a joystick [5,6,34,36], a stylus [2,4,10,12,21,23,24,28,30,35,52], or a robotic manipulator [42,53]; by tracking the motion performed by the human body [7,8,9,25,26,27,47,48]; or even by measuring the force exerted on a handle or acquiring the myoelectric muscle activations [46,54,55]. The VMA-directed discrepancy between the expected and the visually perceived outcome can be introduced by altering the input action in the form of a translation [10,47,56], a rotation [4,5,10,18,21,22,23,25,26,28,34,46,57,58], or a reversal [2,19,20,36] of the path that is traced or even a set of sinusoidal oscillations on the visual field that is presented [7,8,9,17,27,48].

The employment of virtual reality (VR) leads to more adaptable and customizable synthetic scenarios/environments. In fact, a virtual scenario simplifies the integration between the task that has to be done in the environment and the subject’s motor command. On the other hand, a fully virtual scenario allows the design of a large variety of perturbation types that can also be modulated and changed without any restrictions during the experimental protocol [27]. Furthermore, it is well known that a VR-based serious game (SG) amplifies the individuals’ engagement, which should be ensured since participants could stop feeling motivated because of the repetitiveness that is typical of scientific experiments [59,60]. VR can also provide a greater sense of embodiment by mapping human body parts more realistically [61], and this feeling may even be increased by resorting to a full-body avatar representation [62].

The majority of works implementing a framework for VMA have entailed the upper limbs in order to execute either a reaching or a drawing task. Some researchers have employed a screen and a mirror to project the virtual scenario on a plane that is parallel to the physical workspace, in which an external grabbed object, e.g., a joystick, a stylus, or a robotic manipulandum, is exploited to control the cursor correspondent to the hand motion [5,22,38,40,42]. Alternatively, other studies have displayed the trajectory followed by a cursor directly on a screen; this is utilized to allow subjects to visualize the path they trace in the real world on a tablet through a stylus [21,30]. To introduce the visual perturbation that elicits adaptation, the cursor’s direction of movement is rotated [22,23,40,42,52] or even gained [38].

However, such VR-based frameworks can be made to be more motivating by representing the human body in a more realistic way. For this purpose, Lin et al. mapped real movements as those of two hand-held controllers, which are exploited to interact within the VE that is shown through an HMD; in addition, they included either a consistent or a reverted mapping between the real and virtual paths followed during a reaching task [63]. On the other hand, Cristella and colleagues proposed three SGs requiring the participant to perform trunk inclination, elbow flexion/extension, and forearm supination/pronation; they also implemented coherent or reversed correspondence between the motor command in the real world and the visual feedback [36]. These are only some of the several studies targeting VMA during upper-limb motor tasks, whereas few works have proposed workflows stimulating VMA during locomotion activity. For instance, Kannape and colleagues set up a motion tracking area in which subjects have to perform a reaching walking task; further, a screen is employed to show a whole-body avatar in a virtual room, and angular deviation is applied to the captured position to stimulate VMA [25,26].

In view of the above-mentioned works, a paucity in the literature has been noticed about the implementation of a VMA-oriented SG that alters the difficulty of exploring a virtual environment with a whole-body avatar guided by walking steps that are performed in the real world. In this context, the goal of this article is to present the design, development, and test of a framework that aims to evaluate VMA during a locomotion activity by controlling a full-body embodied avatar in a VR scenario (shown on a 2D monitor); the subject has to collect objects along a predefined path. More specifically, the workflow is endowed with an SG that promotes the participants’ engagement by means of mapping perturbations that elicit VMA. Further, a set of quantitative metrics and scores is proposed as performance indicators of the task. A customized experimental protocol is tested with thirteen healthy subjects to evaluate the usability and safety of the proposed system and to test the efficacy of the designed framework; it includes scores for encoding the task difficulty perceived by the subject.

The paper is organized as follows: Section 2 describes materials, i.e., the VMA-oriented framework, which includes a body-tracking system for data acquisition, a treadmill for performing locomotion, an SG for promoting the participant’s engagement, and a calibration stage for coping with inter-session spatial modifications of the setup and inter-subject differences in gait characteristics; Section 2 also exposes the methodology adopted in this work, which comprises metric extraction and statistical analysis. The outcomes of the experimental study are provided in Section 3 and are discussed in Section 4. Ultimately, Section 5 draws the final remarks about the conducted study and delineates ideas for future works.

## 2. Materials and Methods

This section is articulated in the description of the framework utilized for VMA assessment (Section 2.1) and in its experimental test (Section 2.2).

### 2.1. The Proposed Framework

**Figure 1:**
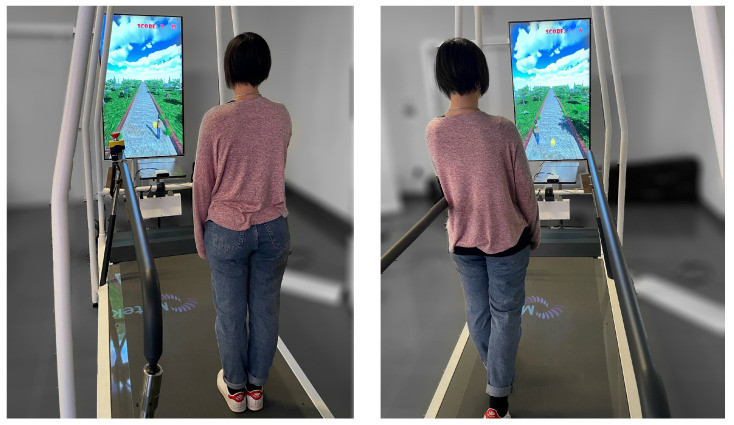
The experimental setup.

The framework that is proposed for experiments eliciting and evaluating VMA is made up of three main components (see Figure 1), which are: a human skeleton tracking system, a treadmill, and a fully customizable VR-based serious game.

The technical requirements and the features of the presented framework were discussed and defined during a number of meetings between the academic authors and the clinical staff of the IRCCS Fondazione Don Carlo Gnocchi ONLUS (Florence, Italy), who expressed the need for a customizable VR-based serious game to evaluate the visuomotor adaptation capabilities of children with neuromotor disorders during locomotion tasks. In particular, the following requirements were defined and used for the design and implementation phases of the presented framework:

- The subject has to control the position of an embodied avatar in a custom virtual environment during a locomotion task;
- The subject has to collect pseudo-random objects that are located on the virtual ground along a path—the avatar can collect objects by hitting them;
- The subject has to walk on a treadmill while collecting the virtual objects, and the treadmill must allow mediolateral (ML) movement of the entire body in order to pick up objects that are positioned on the sides of the path;
- The subject must also experience two kinds of perturbation that alter the position mapping between the real ML position of the subject and the ML position of the avatar in the VE, i.e., GAIN and REVERSAL, that aim to amplify and reverse, respectively, the avatar’s movement respectively;
- The use of a marker-less solution to track the human skeleton body is preferred in order to speed up the experimental setup phase;
- Motivational soundtracks should be used.

#### 2.1.1. The Subject Skeleton Tracking System

**Figure 2:**
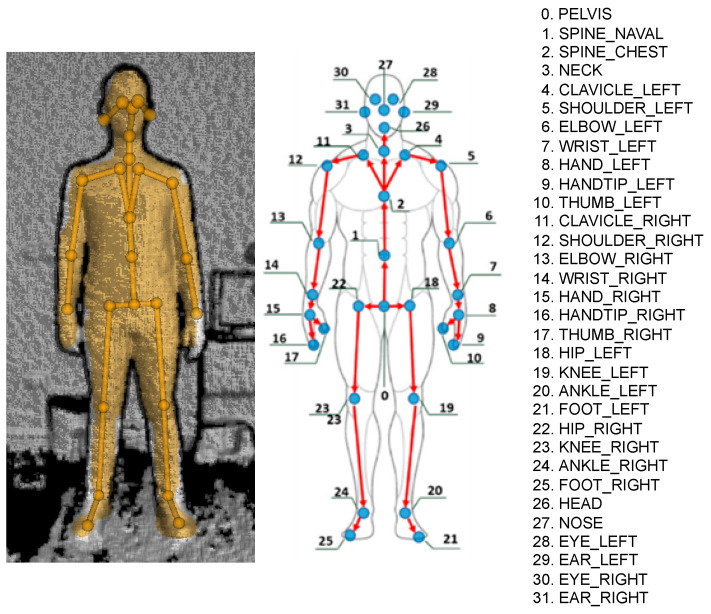
Skeleton tracked by the Azure Kinect SDK.

The first subsystem of the proposed framework is a real-time body-tracking system that is used to let the participant control the avatar’s position within the virtual environment and the modeled articulation joints. In this study, the authors used a 3D Azure Kinect that was positioned 0.5 m away from the subject in order to acquire the scene and the whole front part of the subject’s body. In particular, the Azure Kinect Body Tracking SDK was used to automatically extract the human skeleton, i.e., the poses of all modeled joints and links of the skeleton (see Figure 2), with a frame rate of 50 Hz [64]. It is worth mentioning that the high usage of the Microsoft Kinect in clinical setups during the last decade is mainly motivated by its low price and its high accuracy considering the cost [65,66,67,68].

#### 2.1.2. The Treadmill

A treadmill was chosen as the locomotion surface since it allows walking continuously, thus keeping subjects focused on the task to execute. The treadmill width has to be such that a comfortable walking area allows mediolateral movements for the participants during the whole session. In this specific study, the employed treadmill was the C-Mill VR (by Motek^®^, Amserdam, The Netherlands). It is equipped with a safety frame and two adjustable handrails to prevent subjects from falling (e.g., due to loss of balance) and also provides the possibility to measure some gait parameters that can be exploited to tailor the locomotion to the individual characteristics. It is worth mentioning that the proposed framework is independent of the type of treadmill adopted. Therefore, even a simple, cheap treadmill that is not able to compute the position of the subject’s center of gravity and make it available in real-time can be employed.

#### 2.1.3. The Serious Game

According to the defined requirements, the designed and developed SG allows a subject that is walking on a treadmill to control the position of an embodied avatar with the aim of collecting objects along a path. Specifically, the subject can pick up an object, which may be located either at the center or at the sides of the virtual road, by controlling the ML position of the avatar. The subject can thus translate the avatar along the ML axis by moving his/her body on the treadmill along the real ML plane within a feasible range of motion (RoM). In more detail, the ML position of the subject’s pelvis is used to directly control the ML position of the avatar’s pelvis.

The SG, which was developed using the Unity3D framework (release 2020.3.27f1, Unity Technologies, San Francisco, CA, USA), also implements the link between the human joints tracked by the Kinect Azure SDK (version 1.4.1, Microsoft, Redmond, WA, USA), and the corresponding avatar’s joints by using a Unity 3D demo (GitHub Commit Number: d87e80a, Microsoft, Redmond, WA, USA), which available on the Microsoft GitHub webpage [69].

The game permits placing a series of rewarding objects (e.g., candy) on a straight virtual road in any position. The software also enables collecting these targets by moving a full-body avatar on the virtual endless walkway while walking on the treadmill. Further, a trial is associated with each object, the collection of which entails the game score update. The objects are rendered one at a time and stay visible until they are collected or surpassed by the avatar while advancing in the scene. Subsequently, in order to adapt the difficulty of the task to the specific anthropometric characteristics of each subject, the distance in the real world between two subsequent objects is set equal to eight strides. In addition, the SG continuously regenerates the virtual road with the aim of rendering the endless walkway that corresponds to the treadmill in the real world. It also makes the avatar advance in the scene along the locomotion direction by linearly incrementing the avatar’s position based on a quantity that depends on the subject’s velocity.

**Figure 3:**
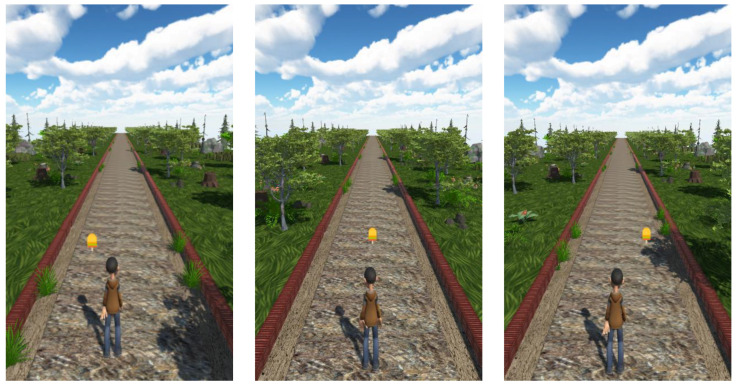
Different screenshots of the main scenario of the serious game.

In this work, the virtual environment was shown on a 2D monitor that was positioned in front of the subject. The screenshots in Figure 3 depict the avatar moving on the virtual road and the target to be picked that are shown within the VE.

Furthermore, proper mapping between real and virtual displacements enables the boundaries in the virtual space to correspond to those of the real workspace, which is defined by the participant’s range of motion along the ML axis. Such a basic mapping constitutes the game functioning in the absence of perturbations.

**Figure 4:**
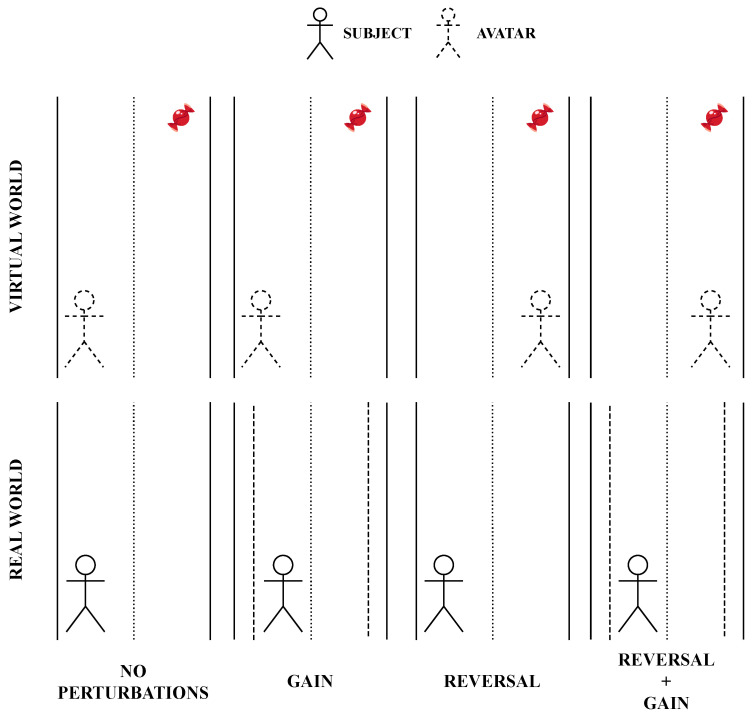
Graphical representation of the effects of the four positional mapping conditions. Vertical continuous lines indicate the boundaries of the treadmill and the virtual road, whereas the dashed lines refer to the sides of the reduced real space due to the gain factor. The red candy represents the virtual object that has to be collected.

Concerning the possible visual perturbations that might characterize a single trial, the authors decided to allow the manipulation of the mapping between the ML position of the avatar on the virtual road and the ML position of the subject on the treadmill. In particular, four possible mapping conditions between the real and virtual environments were implemented (see Figure 4) and are listed below:

- *No Perturbations*: the mapping is not altered—the avatar moves as the subject—when the subject reaches the side of the treadmill, the avatar is on the side of the virtual road;
- *Gain*: the mapping is altered—the avatar moves following the same sense of the subject’s movement—the avatar’s movements on the ML plane are amplified by a specific gain factor;
- *Reversal*: the mapping is altered—the avatar moves following the opposite sense of the subject’s movement (i.e., when the subject moves to the right, the mapping leads the avatar to the left, and vice versa)—when the subject reaches the left side of the treadmill, the avatar is on the right side of the virtual road, and vice versa;
- *Reversal + Gain*: the mapping is altered—the above-mentioned perturbations are simultaneously applied, thus amplifying and reverting the avatar’s position at the same time.

Note that due to the gained mapping, the avatar may go beyond the limits of the road when the subject reaches the treadmill boundaries. Hence, the SG guarantees that the avatar is always visible thanks to saturation that is imposed on its lateral position. The SG has been implemented as a sequence of several scenes, each of which is provided with a soundtrack that aims to keep subjects motivated and to prevent them from getting bored or losing motivation. Finally, it is worth reporting that the implemented SG also provides a user-friendly form that allows the clinical staff to build a customized experimental protocol, i.e., the specific sequence of objects to collect and the type of perturbation that can be applied within specific phases of the protocol.

#### 2.1.4. Initial Calibration Procedures

A set of initial calibration procedures has been also designed both for adapting the task to the specific anthropometric characteristics of the subject and for managing the unknown pose of the Azure Kinect reference frame and treadmill reference frame. As a first step, the following three parameters are acquired with dedicated tests and inserted within a specific form of the SG concerning the subject data:

- Preferred walking speed (PWS): the treadmill speed is kept fixed and is equal to the PWS during the entire experimental session—such a speed is also used to translate the avatar’s center of mass along the locomotion direction—this speed is experimentally found with the help of the clinical staff by gradually increasing the belt speed until the participants report that they are walking at their PWS [70,71];
- Mean step length: this parameter is used to define the distance between two subsequent objects to collect—in this specific work, the step length was automatically extracted by the C-Mill software (CueFors 2.2.3, Motek Medical B.V., Vleugelboot 14, 3991 CL Houten, Netherlands), even though any other solution based on skeleton data processing can be used;
- Range of motion of the subject’s pelvis on the treadmill along the mediolateral axis—such a measure is used to adapt the real subject’s RoM to the avatar’s RoM.

**Figure 5:**
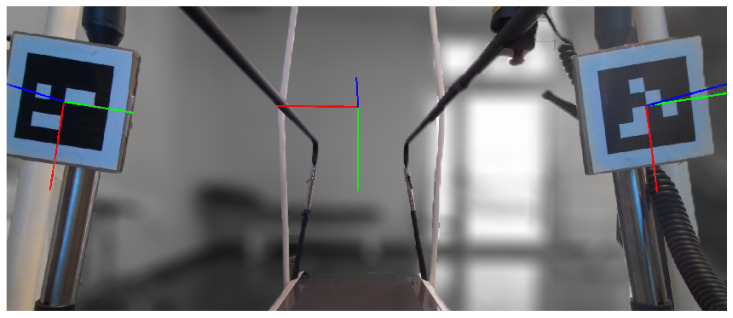
A processed frame acquired by the Azure Kinect that shows the two ArUco markers positioned on the lateral handrails of the treadmill and a 2D representation of both the markers and the treadmill reference frames. Red, green and blue lines represent the x-axis, y-axis and z-axis of the reference frames, respectively.

As a second step, a procedure that is able to define the relative pose between the Azure Kinect camera and the treadmill is executed since it is necessary to correctly map the real and virtual worlds. The authors designed a procedure that makes use of ArUco, an open-source library that allows defining and tracking 2D markers [72]. As shown in Figure 5, two different ArUco markers are positioned on the two lateral handrails of the treadmill so that they are both visible to the Azure Kinect that is capturing the entire scene. For each marker, the ArUco library is able to compute the pose of the relative reference system with respect to the camera reference system. Finally, the final reference system of the treadmill is defined as follows: it is positioned at the midpoint of the origins of the two marker reference systems, and its x-axis is oriented as the line that crosses the two origins, whereas the y-axis is oriented as the gravity vector.

#### 2.1.5. Performance Metrics

In addition to the system presented above, the proposed framework also considers a set of metrics that can be used to quantitatively evaluate the performance of each subject. The percentage of collected targets (CT) is an obvious metric that is examined to evaluate the visuomotor adaptation capability. However, the CT metric does not encode any information concerning the trajectory that the subject followed for each target. Hence, for each object to be collected, three metrics are computed to quantify three different characteristics of the 2D trajectory of the avatar’s pelvis. In more detail, the metrics consider the avatar’s trajectory between its 2D position at the end of the previous trial and its 2D position at the end of the current trial, i.e., when the avatar either hits or surpasses the object:

- Normalized Path Length (NPL): the length of the actual path divided by the length of the minimum length path (MLP), which is the straight line that passes through 2D points, i.e., start and end positions;
- Normalized Area (NA): the area between the actual path and the MLP divided by the MLP length;
- Initial Angle Error (IAE): the angle between the MLP and the segment joining the avatar’s initial position with the point of the real path that corresponds to the first peak of the distance from the MLP.

**Figure 6:**
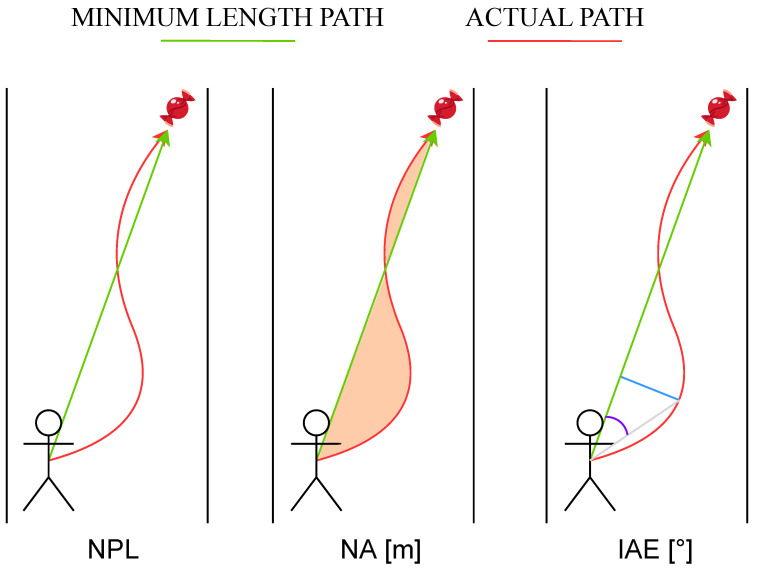
Kinematic-based features selected for assessing difficulty modification: NPL is the ratio between the length of the actual path and the one of the MLP; NA measures the area between the actual path and the MLP divided by the length of the MLP; IAE is the angle between the MLP and the segment joining the avatar’s initial position with the point of the actual path that corresponds to the first peak of the distance from the MLP. The purple line depicts the IAE angle, which is the angle between the MLP segment and the grey line; this latter line represents the segment joining the avatar’s position at the beginning of the trial with the point of the actual path that corresponds to the first peak of the error signal along the path. Such error signal is the length of a segment (blue line) that joins the MLP and the actual path and is perpedicular to the minimum path.

In view of the definitions of the trajectory-based features, it is inferable that the higher their values, the worse the performance. The meaning of the extracted kinematic-based metrics is graphically explained in Figure 6.

### 2.2. Framework Test

The framework test is intended to prove the quality of the game development and the features extracted within the post-processing phase in terms of capturing the task difficulty related to the mapping perturbations. Moreover, the proposed workflow was tested from the point of view of usability and safety, which are important requirements to be met in a clinically oriented context. For this purpose, this work involved healthy subjects as participants and included an experimental protocol to be followed for correct task execution. Furthermore, the proposed features, which encode the outcomes obtained in the different mapping conditions, were compared and statistically analyzed.

#### 2.2.1. Participants

Thirteen healthy children (10.2 ± 1.7 years old, six males) were recruited from the IRCCS “Don Carlo Gnocchi”. Inclusion criteria were: (1) children who exhibited neither neurological nor cognitive disorders (such conditions were verified through two batteries of neuropsychological tests, which are WISC-IV and NEPSY-II [73,74]), (2) children that usually spent between one and three hours per week playing video games. Each subject was informed about the task execution and the game details without mentioning any information about the visual perturbations in order to prevent results from being influenced by this awareness.

#### 2.2.2. Experimental Protocol

The experimental protocol was arranged with the clinicians in such a way as to make the SG suit the subject’s characteristics, thus being more realistic in terms of gait simulation. Hence, prior to the game session, subjects’ parameters, which are the preferred walking speed, the step length, and the range of motion along the ML axis, were measured by means of the sensors embedded in an instrumented treadmill (C-Mill VR by Motek^®^). It is worth noting that if the setup is based on a standard non-instrumented treadmill, such subject parameters can be derived by analyzing the 3D data of the human skeleton extracted by the Kinect Azure.

**Figure 7:**
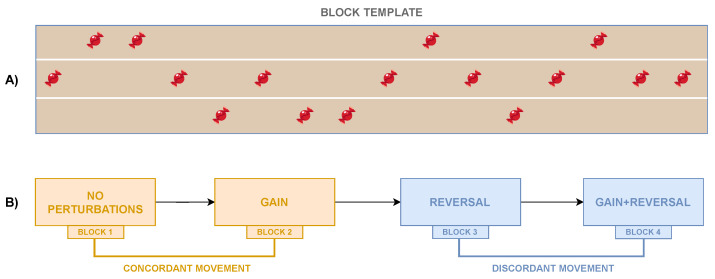
(**A**) Arrangement of the targets (e.g., candy) in each block. Sixteen objects are placed on the virtual road such that the quantity on the right side is the same as that on the left side, thus preventing the avatar from direct movements between the two extremes. (**B**) Sequence of the four blocks defined by the experimental protocol and associated with the mapping conditions explained in Section 2.1.3.

The authors proposed a customized experimental protocol to test the presented framework, which is detailed just below; however, multiple experimental protocols featuring different target sequences, different target positions, or different sequences of perturbations might be used according to the specific needs. In this specific test study, the author defined a template block (see Figure 7A) that considers a predefined sequence of targets that are positioned at the center or the sides of the virtual road. The used sequence was chosen to ensure that the number of targets on the right side was equal to the number of the ones on the left side, and the avatar must not directly move between the two road sides. It is worth being reminded that the targets were rendered one by one so that the subject was fully focused on just one target at a time, thus avoiding any anticipatory effect.

Given the definition of a block, the experimental protocol consists of four subsequent blocks, each of which is characterized by a different mapping condition. As shown in Figure 7B, the defined sequence of the conditions is as follows: (1) *No Perturbations*, (2) *Gain*, (3) *Reversal*, and (4) *Reversal + Gain*. The authors are aware that the fixed order of succession of the conditions does have an impact on the results due to the learning effect and that the specific tested experimental protocol has not been properly designed for a standard motor control/learning study. In fact, the objective of the study is to propose a system that can be used to assess visuomotor adaptation capabilities and verify that the introduced perturbations produce some effects that can be captured by the performance metrics. More specifically, the avatar’s motion on the ML plane occurs in the same sense (concordant) as the real one in the first two blocks, whereas its lateral movements occur in the opposite sense (discordant) with respect to the participant’s ones in the last two blocks. In the first block, no perturbation is included, thus making the lateral movement of the virtual character occur in the same direction and with no amplification with respect to the real displacement; in the second block, the avatar’s position along the ML axis is amplified such that the needed real range of motion is reduced by 25%. The third block is characterized by the *Reversal* condition, and, finally, in the last block, the *Reversal + Gain* condition is applied using the same gain factor as the second block.

#### 2.2.3. Comparisons and Statistical Analysis

**Figure 8:**
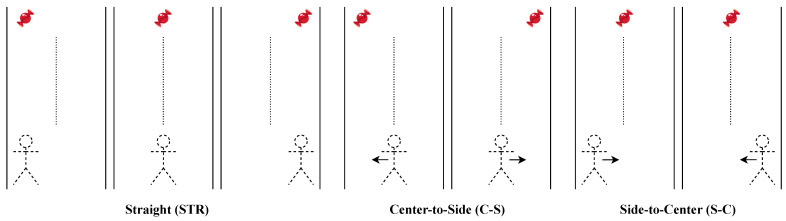
Three directions can be recognized for the line connecting two consecutive targets: for accomplishing the collection task, the avatar can either keep the direction in the straight (STR) case, move from the center to one of the sides in the center-to-side (C–S) case, or move from one of the sides to the center in the side-to-center (S–C) case. The arrows indicate the direction in which the avatar should move in the game to collect the object; the dashed lines depicts the center of the virtual road; the candy represents the virtual object that has to be collected.

The metrics extracted in the different conditions were compared to ascertain whether they were able to capture the differences among the defined mapping conditions. Moreover, the authors believe that the direction of the minimum length path (i.e., the straight line that passes through the initial avatar position and the target position) is an important factor that can be analyzed, since it might have an impact on the performance. In detail, as shown in Figure 8, three directions may be recognized: straight (STR), center-to-side (C–S), and side-to-center (S–C), where the side may be either left or right. Therefore, the authors performed the following comparisons, considering all subjects:

- Comparison 1—among mapping conditions;
- Comparison 2—among mapping conditions grouped by directions;
- Comparison 3—among directions grouped by mapping conditions.

**Figure 9:**
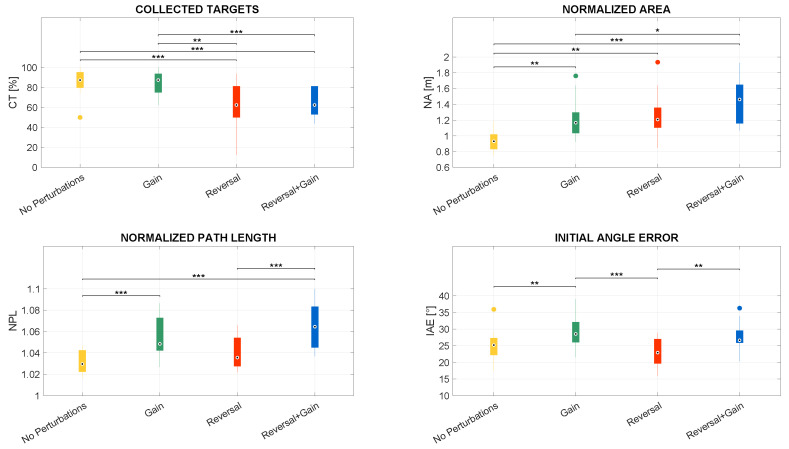
Boxplots of the distributions of the chosen features computed for each mapping condition, with * representing statistically significant comparisons with *p* < 0.05, ** representing statistically significant comparisons with *p* < 0.01, and *** representing statistically significant comparisons with *p* < 0.001. The dots represent the outliers.

**Figure 10:**
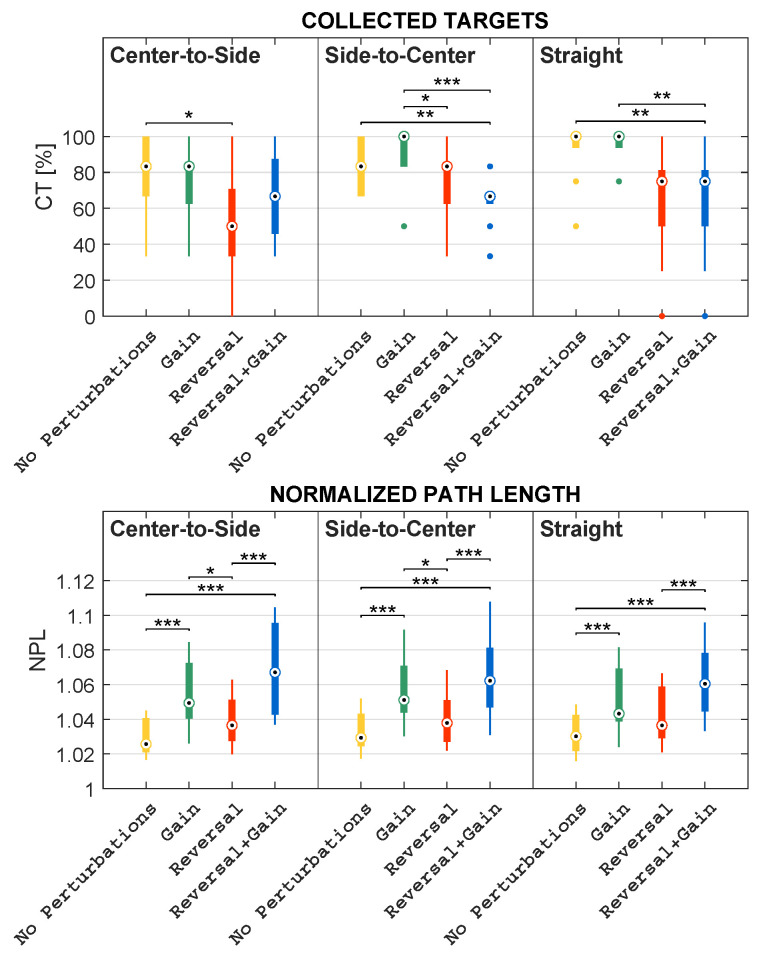
Boxplots of the distributions of the CT and NPL features computed in each mapping condition, grouped by direction, with * representing statistically significant comparisons with *p* < 0.05, ** representing statistically significant comparisons with *p* < 0.01, and *** representing statistically significant comparisons with *p* < 0.001. The dots represent the outliers.

**Figure 11:**
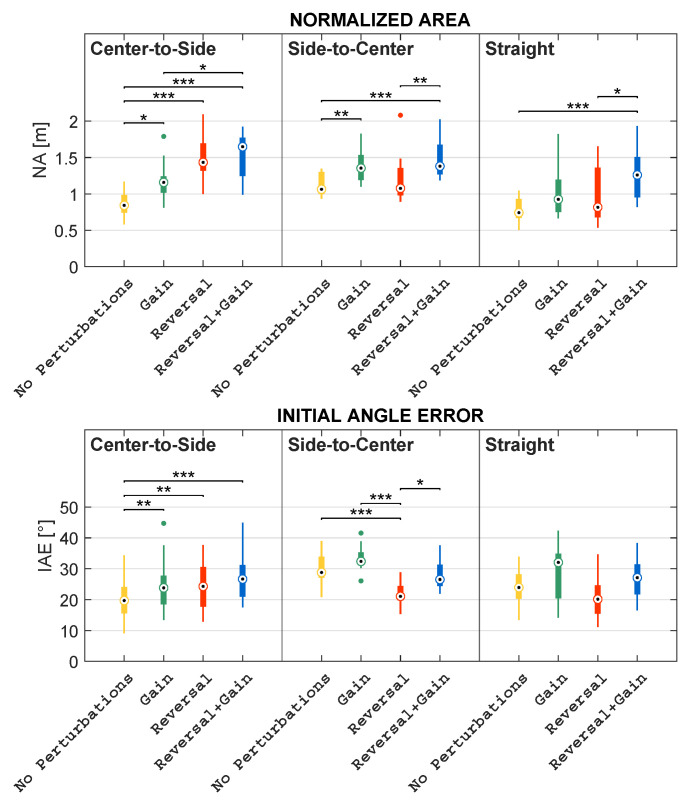
Boxplots of the distributions of the NA and IAE features computed in each mapping condition, grouped by direction, with * representing statistically significant comparisons with *p* < 0.05, ** representing statistically significant comparisons with *p* < 0.01, and *** representing statistically significant comparisons with *p* < 0.001. The dots represent the outliers.

**Figure 12:**
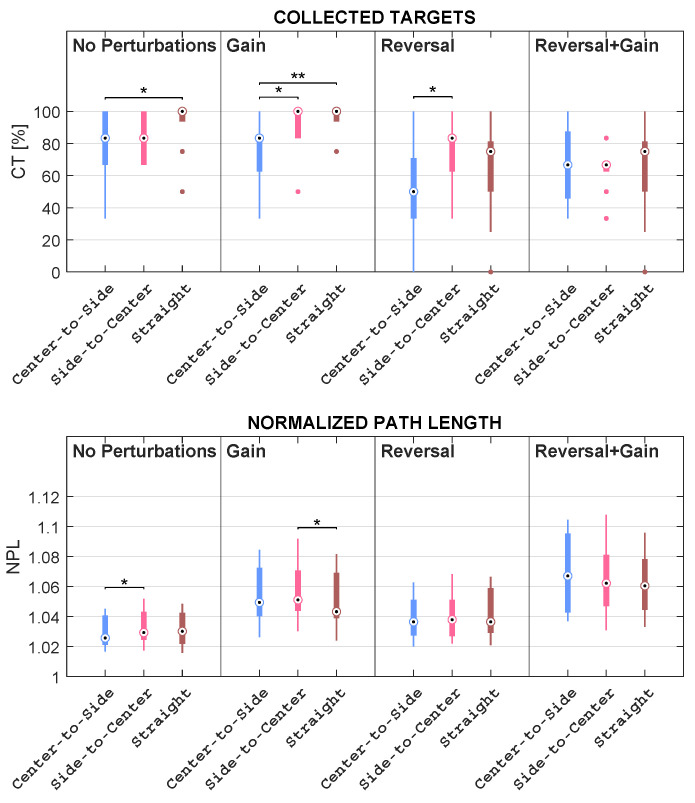
Boxplots of the distributions of the CT and NPL features computed for each direction, grouped by mapping condition, with * representing statistically significant comparisons with *p* < 0.05, and ** representing statistically significant comparisons with *p* < 0.01. The dots represent the outliers.

**Figure 13:**
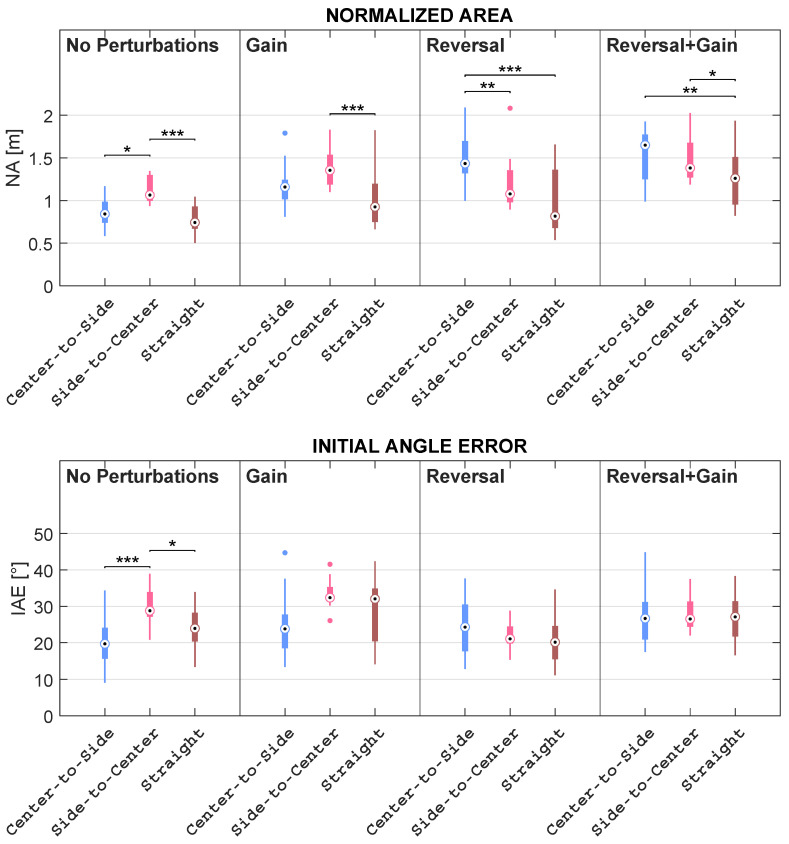
Boxplots of the distributions of the NA and IAE features computed for each direction, grouped by mapping condition, with * representing statistically significant comparisons with *p* < 0.05, ** representing statistically significant comparisons with *p* < 0.01, and *** representing statistically significant comparisons with *p* < 0.001. The dots represent the outliers.

Concerning Comparison 1, for each subject, the mean of kinematic features and the percentage of collected objects were computed within the mapping condition; regarding Comparison 2, for each subject, the mean of kinematic features and the percentage of collected objects were computed within the mapping condition considering each direction independently; in addition, as regards Comparison 3, for each subject, the mean of kinematic features and the percentage of collected objects were computed within the direction considering each mapping condition independently. These comparisons are pictorially depicted in the boxplots reported in Figure 9, Figure 10, Figure 11, Figure 12 and Figure 13.

After that, statistical comparisons were performed with the non-parametric Friedman’s test since the hypothesis of a Gaussian distribution is excluded by the limited number of children involved in this study. When a significant difference was found in this way, a deeper analysis was performed through a pairwise post-hoc test with Bonferroni’s correction with a significance level set to *p* < 0.05. All the analyses were conducted using MATLAB 2021b.

## 3. Results

This section presents the results of the twofold test that the authors performed for the proposed framework: the outcomes concerning the usability, safety, and feasibility in a clinical context are reported in Section 3.1, whilst the results about the efficacy of the designed features in capturing the difficulty modification due to mapping perturbations are described in Section 3.2.

### 3.1. Feasibility in a Clinical Context

The proposed framework targeting VMA meets different feasibility requirements such that it is usable in a clinical context. First, setting up an experimental session is not excessively time-consuming, since very few and fast calibration procedures are needed and, particularly, a marker-less human-skeleton tracking system is employed. It is well known that clinical experimental sessions should also be characterized by fast procedures because it is important to keep participants interested and motivated during the entire experimental procedure.

Moreover, the user-friendliness of the software is guaranteed by offering a protocol that is simple to follow for clinicians. Another aspect that plays an important role is related to the possibility of defining protocols that are tailored to the individual anthropometric characteristics, thus aiming to adapt the game difficulty to the specific subject and allowing for inter-subject comparisons.

Finally, the proposed framework has been proven to ensure children’s safety, since neither sickness (e.g., vertigo) nor loss of balance were reported by the participants; nevertheless, the risk of falls due to the potential loss of balance is prevented by means of the support components with which the treadmill is equipped.

### 3.2. Quantitative Metric Validation

This subsection describes the results obtained concerning the comparisons listed in Section 2.2.3. In more detail, the authors compared mapping conditions, mapping conditions grouped by directions, and directions grouped by mapping conditions. Such comparisons are illustrated in the boxplots reported in Figure 9, Figure 10, Figure 11, Figure 12 and Figure 13.

#### 3.2.1. Differences among Mapping Conditions

Statistically significant differences were revealed for each feature when comparing mapping conditions, regardless of the direction. The outcomes of the proposed metrics for evaluating difficulty alteration among mapping conditions are pictorially depicted in the boxplots reported in Figure 9.

CT (Collected Targets) is the percentage of the objects collected by the avatar. In this regard, Friedman’s test reveals that the CT significantly differs among mapping conditions, with *p* < 0.001. Further, according to post-hoc tests, the CT in the conditions with reverted mapping is significantly lower than the CT in both mappings without reversal; instead, no statistically significant differences were found in the CTs between the two conditions without reversal, as well as between the two mappings including reversal. In fact, the CT in *Reversal* is significantly lower than the one in *No Perturbations*, with *p* < 0.001, and the one in *Gain*, with *p* < 0.01. Similarly, the CT in *Reversal + Gain* is significantly lower than the one in *No Perturbations* and the one in *Gain*, with *p* < 0.001 in both cases.

Remembering that NA (Normalized Path Integral Error) is a normalized measure of the area between the actual path and the length of the MLP, Friedman’s test showed statistically significant differences in NA among mapping conditions, with *p* < 0.001. Post-hoc tests revealed the mapping without perturbations is significantly different from the mappings including either one or two perturbations: in particular, the NA in *No Perturbations* is significantly lower than those in *Gain* and *Reversal*, with *p* < 0.01, and the one in *Reversal + Gain*, with *p* < 0.001. In addition, the NA in *Gain* is significantly lower than the NA in *Reversal + Gain*, with *p* < 0.05.

As concerns NPL (Normalized Path Length), which is the length of the path actually covered by the avatar divided by the length of the MLP, Friedman’s test led to statistically significant differences in NPL, with *p* < 0.001 among mapping conditions. Remarkably, in the mapping conditions characterized by *Gain*, the NPL is significantly higher than the NPL observed within the conditions without *Gain*: in fact, the NPL in *Gain* is significantly higher than the one in *No Perturbations* as well as the one in *Reversal + Gain* is significantly higher than the one in *Reversal*, with *p* < 0.001 in both cases. The NPL in *Reversal + Gain* is also significantly higher than the one in *No Perturbations*, with *p* < 0.001, whilst no statistically significant differences were reported between the NPL in *Gain* and the one in *Reversal*.

With regards to IAE (Initial Angle Error), which indicates the initial angular deviation from the MLP direction, Friedman’s test reveals that the IAE significantly differs among all conditions, with *p* < 0.001. The IAE trend among the tested conditions is very similar to the NPL trend. The IAE measured in the mapping conditions characterized by *Gain* is higher than the IAEs observed within the conditions without *Gain*: the IAE in *Gain* is significantly higher than the one in *No Perturbations*, and the IAE in *Reversal + Gain* is significantly higher than the one in *Reversal*, with *p* < 0.01 in both cases. Further, the IAE in *Reversal* is significantly lower than the one in *Gain*, with *p* < 0.001.

#### 3.2.2. Differences among Mapping Conditions Grouped by Directions

The comparisons presented above are useful to provide a general overview of the differences that have been observed among the different mapping conditions. However, a deeper analysis is necessary and has been performed to investigate the role of the different MLP directions (see Figure 8). In particular, in this section, the authors present the results obtained when comparing the different conditions focusing on each of the three possible directions: straight (STR), center-to-side (C–S), and side-to-center (S–C). The results of these comparisons are pictorially depicted in the boxplots reported in Figure 10 and Figure 11.

Friedman’s test revealed that CT significantly differs among mapping conditions within all directions, with *p* < 0.01. Within each direction, the trend in differences among the mapping conditions is almost similar both to each other and to the differences observed in the general comparison (Section 3.2). In fact, it turned out that the CT in *No Perturbations* is higher than the CT in *Reversal + Gain* in all directions, but this difference is statistically significant only within the STR and the S–C directions, with *p* < 0.01. Similarly, the CT in *Gain* is higher than the one in *Reversal + Gain*, but this comparison is statistically significant only in the STR and S–C directions, with *p* < 0.01 and *p* < 0.001, respectively. On the other hand, the CT in *Reversal* is lower than the one in *Gain* in all directions, but this relation is statistically significant only in the S–C direction, with *p* < 0.05. Moreover, the CT in *Reversal* is always less than the one in *No Perturbations*, but this is statistically significant only in the C–S direction, with *p* < 0.05.

Concerning the NPL, it is worth noting that, within each direction, the relative differences among the mapping conditions are almost identical both to each other and to the differences observed in the general comparison (Section 3.2). Friedman’s test proved that this metric is significantly different among mapping conditions, with *p* < 0.001 within all directions. More specifically, within all directions, the NPL in *No Perturbations* is proven to be significantly lower than the one in *Gain*, and the NPL in *Reversal* is significantly lower than the one in *Reversal + Gain*, with *p* < 0.001 in both cases. In addition, post-hoc tests also revealed that the NPL in *No Perturbations* is significantly lower than the one in *Reversal + Gain*, with *p* < 0.001 within all directions. On the other hand, the NPL in *Reversal* is higher than the one in *No Perturbations* within all directions, but this relation is not statistically significant in any direction. Similarly, the NPL in *Gain* is higher than the one *Reversal* within all directions, but significant differences are yielded only within the C–S and S–C directions, with *p* < 0.05 in both cases.

Concerning NA, Friedman’s test also showed significant differences in NAs among mapping conditions within all directions, with *p* < 0.001. The relative differences among conditions of *No Perturbations*, *Gain*, and *Reversal + Gain* are similar both to each other and to the differences observed in the general comparison (Section 3.2). The main observed difference concerns the NA values measured during *Reversal*: in S–C and STR, the values are comparable to those of *No Perturbations*, whereas in C–S, the NA values are higher than both the *No Perturbations* and *Gain* values. In more detail, the NA in *No Perturbations* is significantly lower than the one in *Reversal + Gain* within all directions, with *p* < 0.001; the NA in *No Perturbations* is also lower than the one in *Gain* in all directions, but this difference is statistically significant only within the C–S and S–C directions, with *p* < 0.05 and *p* < 0.01, respectively. Furthermore, the NA in *Reversal* is significantly higher than the one in *No Perturbations* only within the C–S direction, with *p* < 0.001, whilst their medians are almost the same in the remaining directions. However, the NA in *Reversal* is always lower than the one obtained in *Reversal + Gain*, although this difference is statistically significant only within the S–C and STR directions, with *p* < 0.01 and *p* < 0.05, respectively.

Ultimately, regarding the IAE values, it is worth noting that the relative differences among the conditions in the S–C and STR directions are analogous both between these two directions and with respect to the general comparison (Section 3.2). Differently, the IAE values of *Reversal* and *Reversal + Gain* registered in C–S are comparable with values acquired in *No Perturbations* and *Gain*. According to Friedman’s test, the IAE significantly differs among mapping conditions within all directions, with *p* < 0.001. Nonetheless, post-hoc tests lead to statistically significant differences only within the C–S and S–C directions. In fact, the IAE in *No Perturbations* is always lower than the one in *Gain*, but this difference is statistically significant only within the C–S direction, with *p* < 0.01. On the other hand, the IAE in *Reversal* always lessens the one in *Reversal + Gain*, but a statistically significant difference is yielded only within the S–C direction, with *p* < 0.05. Furthermore, the IAE in *Gain* is higher than the one in *Reversal* within the S–C direction, with *p* < 0.001, whereas no statistically significant differences resulted from the remaining directions. Similarly, the IAE in *No Perturbations* is either significantly lower or higher than the one in *Reversal* within the C–S and S–C directions, with *p* < 0.01 and *p* < 0.001, respectively.

#### 3.2.3. Differences among Directions Grouped by Mapping Conditions

In this section, the results related to the comparisons among the three directions for each specific mapping condition are presented (see Figure 12 and Figure 13). The comparisons presented in the previous subsection allowed for scrutinizing the dependence between the differences among the mapping conditions and the directions. The comparisons presented in this section allow for directly investigating the role of the direction given a specific mapping condition.

Friedman’s test revealed that CT does not significantly differ among the directions within *Reversal + Gain*, whereas it does happen for all the remaining conditions, with *p* < 0.05. Furthermore, the CT in the C–S direction is always lower than the one in the STR direction within all conditions, but this relation is statistically significant only within *No Perturbations* and *Gain*, with *p* < 0.05 and *p* < 0.01, respectively. On the other hand, the CT in the STR direction is higher, but not significantly, than the one in the S–C direction only within *No Perturbations* and *Reversal + Gain*, whilst the CT median in the STR direction is either equal to or lower than the one in the S–C direction within *Gain* and *Reversal*, respectively. Similarly, the CT in the C–S direction significantly lessens the one in the S–C direction within *Gain* and *Reversal*, with *p* < 0.05 in both cases, whereas the medians in the C–S and S–C directions are the same within *No Perturbations* and *Reversal + Gain*.

When focusing on the NPL, it mainly emerges that this feature does not depend on the direction, with very few but not marked exceptions. In fact, the only significant findings are: the NPL in the S–C direction is significantly higher than both the one in the C–S direction within *No Perturbations* and the one in the STR direction within *Gain*, with *p* < 0.05 in both cases.

With regard to NA, Friedman’s test reported statistically significant differences among directions within all conditions, with *p* < 0.001. Two different behaviors emerged when comparing the mapping conditions without reversal with the mapping conditions characterized by the reversal. Specifically, the NA values measured in S–C are higher than the values acquired in the other two directions in both *No Perturbations* and *Gain*. For what concerns *Reversal* and *Reversal + Gain*, it turned out that the NA values in C–S are higher than the NA values in S–C, which, in turn, are higher than the NA values acquired in STR. In more detail, the NA in the C–S direction is higher than the one in the STR direction within all conditions, even if this relation has proven to be statistically significant only within *Reversal* and *Reversal + Gain*, with *p* < 0.001 and *p* < 0.01, respectively. Analogously, the NA in the STR direction lessens the one in the S–C direction within all conditions, although this difference is statistically significant only within *No Perturbations* and *Gain*, with *p* < 0.001, as well as within *Reversal + Gain*, with *p* < 0.05. The NA in the S–C direction is also higher than the one in the C–S direction within *No Perturbations*, with *p* < 0.05, and *Gain*, with no statistically significant difference; instead, the NA in the S–C direction significantly lessens the one in the C–S direction within *Reversal*, with *p* < 0.01, and within *Reversal + Gain*, though with no statistically significant difference.

Ultimately, the IAEs proved by Friedman’s test to be significantly different among directions only within *No Perturbations*, with *p* < 0.001; post-hoc analysis revealed statistically significant differences among directions within *No Perturbations*. In particular, the IAE values in the C–S direction are lower than the ones in the S–C direction within *No Perturbations*, with *p* < 0.001, and within *Gain*, though with no statistically significant differences; on the other hand, the median in the C–S direction is either slightly higher or equal with respect to the one of the S–C direction within *Reversal* and *Reversal + Gain*, respectively. Similarly, the IAE in the C–S direction is lower than the one in the STR direction within *No Perturbations* and *Gain*, although not significantly, whereas the IAE median in the C–S direction is either slightly higher or equal with respect to the IAE median in the STR direction within *Reversal* and *Reversal + Gain*, respectively. Furthermore, the IAE in the STR direction is lower than the one in the S–C direction within *No Perturbations*, with *p* < 0.05, as well as within *Reversal*, although with no statistically significant difference; on the other side, the IAE median in the STR direction is equal to the IAE median in the STR direction within *Gain* and *Reversal + Gain*.

## 4. Discussion

In this work, the authors present a VR-based framework that was designed and implemented to evaluate visuomotor adaptation capabilities during locomotion tasks. The experimental protocol considers a subject who is asked to directly control the position of an embodied avatar in a custom virtual world with the goal of collecting random objects that appear along a predefined path. The real locomotion task is performed on a treadmill, which allows translation of the subject’s pelvis along the mediolateral axis. The positional mapping between the subject and the avatar can be altered in specific phases of the game to assess the adaptation abilities of the subject by introducing two kinds of perturbation: amplified and reversed movement of the avatar with respect to the subject’s. The proposed framework also integrates a set of scores/metrics that can be used to quantitatively evaluate how well the task has been performed in each phase of the game. Such metrics are the percentage of collected targets (CT) and three variables extracted from the trajectories covered by the avatar during the game: the normalized path length (NPL), the Normalized Area (NA), and the Initial Angle Error (IAE). It is worth mentioning that the higher the value of any trajectory-based feature, the worse the performance and the more challenging the exploration task.

Thirteen healthy children were recruited to preliminarily test the proposed system and validate the designed set of metrics. Each subject was asked to collect 64 objects placed in a pseudo-random position along the path. As described in Section 2.2.2, four different kinds of mapping were tested during each session: *No Perturbations*, *Gain*, *Reversal*, and *Reversal + Gain*. During the experimental sessions, the framework proved to be safe, since participants reported neither any forms of sickness nor had risk of falling. Further, subjects’ motivation was preserved through a quick initial calibration procedure, a marker-less tracking configuration, an intuitive interface for clinicians, and a realistic gait simulation that is tailored to individual motor abilities and anthropometric characteristics.

Concerning the tested experimental protocol, the virtual objects are placed so that the avatar should ideally move in three directions within the virtual road to ensure a path variability, i.e., straight (STR), center-to-side (C–S), and side-to-center (S–C), under the four mapping conditions. In order to evaluate the capability of the visuomotor perturbations in making the task more difficult and to validate the relevance of the proposed features, a set of comparisons was performed, which is listed:

- Comparison 1—among mapping conditions;
- Comparison 2—among mapping conditions grouped by direction;
- Comparison 3—among directions grouped by mapping condition.

The quantitative results related to all comparisons and metrics were presented and reported in detail in the previous subsection. The results surely show that there are statistically significant differences among both the proposed mapping conditions and the different considered directions for all computed metrics. Even though the presented work is not a proper/traditional motor control or motor learning study, the authors have deeply analyzed the obtained results and report the related discussion below. The authors believe that the proposed comparisons and the corresponding argumentations might be useful to analyze the results that would be observed in a study designed to examine human motor control under visuomotor perturbations in either healthy or pathological subjects. Before running all the experimental sessions, the authors hypothesized the following:

- *Reversal* is more challenging than *Gain*, and *Reversal + Gain* is more difficult than *Reversal* and *Gain*;
- The targets positioned along the STR direction are easy to pick, since no movement along the ML axis is required if the previous object has been collected.

Visual perturbations effectively make the exploration task more complicated (see Section 3.2). Analyzing the comparisons among the mapping conditions, it turned out that the “reversal factor” (see *Reversal* and *Reversal + Gain*) had a higher influence on the percentage of collected targets and the normalized area, whereas the “gain factor” worsened the values of normalized path length and initial angle error. These results indicate that the reversal actually makes the target difficult to collect, whereas gain produces several corrections of the trajectory, i.e., higher NPL values, and an initial bigger deviation, i.e., higher IAE values.

When comparing the different mapping conditions within each specific direction (see Section 3.2.2), it emerged that the observed differences for the percentage of collected targets and the normalized path length are similar to the general differences among the values averaged among the directions. Such similarity in terms of relative differences is also confirmed for the normalized area and the initial angle error, with the exception of the Center-to-Side direction. In fact, in this specific direction, the relation between the metric values acquired during the “reversed” mappings and the ones acquired during the “not reversed” mappings are different if compared with the general comparison (see Section 3.2). In particular, the Center-to-Side direction seems to require more effort than the other directions. In the authors’ opinion, the following motivation could explain such results: if the avatar is at the center of the virtual road and the target is on the side, the subject has a higher chance of choosing the wrong direction since he/she can physically move either to the left or to the right; on the contrary, if the avatar is on the side of the road (this means that the subject is on the side of the treadmill) and the target is at the center of the road, the movement allowed by the subject can only be in one sense, because of the physical boundaries, i.e from the side to the center of the treadmill.

The authors also focused on the direct comparison of the values acquired in each direction for each specific mapping condition (see Section 3.2.3). The normalized path length is a metric that is almost independent of the direction of the path towards the target. More specifically, Straight proved to be the direction in which picking up objects is easier than in the other directions, since both CT and NA values in the STR direction are better than the ones acquired in both the C–S or S–C directions within almost all mapping conditions. On the other hand, the collection task was more complicated in the C–S direction than in the S–C direction when a reversal was introduced, since the CT, NA, and IAE in the C–S direction are worse than the ones in the S–C direction within *Reversal*. Interestingly, the angular error obtained in the S–C direction is worse than that in either the STR direction or the C–S direction when no reversal is applied, as IAE in the S–C direction is higher than the one in the remaining directions within *No Perturbations* and *Gain*. This may be due to the fact that subjects tend to align the avatar to the target location as soon as possible, i.e., the center of the road, thus increasing the angular error at the beginning of the trial.

## 5. Conclusions

In this work, the authors presented a framework based on serious games in virtual reality for the evaluation of visuomotor adaptation (VMA) capabilities during a motor task. The latter consists in the control of a whole-body avatar in a visually perturbed virtual scenario while walking on a treadmill. Further, the serious game ensured participants’ motivation during experimental sessions by means of quick calibrations and a realistic gait simulation of the avatar, which is supposed to collect virtual targets pseudo-randomly positioned along the virtual path. All the performed experimental sessions qualitatively proved the feasibility of using the framework in a clinical context, due to the setup safety and the software user-friendliness. In addition, it effectively stimulated visuomotor adaptation through mapping alterations (e.g., gain and reversal of the avatar’s position along the ML axis), as encoded by a set of metrics that is made up of the percentage of collected targets and three features extracted by the avatar’s trajectory. Comparison of the computed metrics in different game conditions demonstrated the efficacy of the implemented visuomotor perturbations and the validity of the introduced metrics in describing the quality of the performed task. Most notably, the results indicate that reversal increases the difficulty of the object collection, whereas gain leads to correcting of the trajectory many times and deviation with a higher angle at the beginning of the trajectory. The main limitation of the study is related to the experimental sample size, which is unarguably small. Such a limitation could be the beginning of a further recruitment campaign aimed at performing future investigations in the field of either motor control or motor learning. Another important limitation of the study is represented by the fixed succession of the mapping conditions, since the results might be biased by the learning effect. Furthermore, the proposed system may be exploited in practical clinical settings since it might offer novel insights into the quantitative assessment of neuromotor disorders. Several objective parameters can be measured and exploited for analyzing gait as well as to corroborate studies that are focused on human upper districts. In addition, newly proposed biomarkers may be correlated with traditional clinical scores that are associated with pathological conditions of either a cognitive or a motor nature. Such deep study could be used to better cluster patients, with the aim of proposing customized and targeted therapies. Therefore, the proposed feature-based approach can pave the way for clinical assessments of pathologies that determine either motor or cognitive impairments: for instance, VMA capabilities can be assessed to study the alteration to the sense of agency in children with cerebral palsy. In so doing, it would be possible to integrate and enhance the conventional rehabilitative programs by giving additional biomarkers and thus propose new solutions to integrate the existing clinical pathways [75]. In addition, an HMD may be used to design balance experiments while offering an even higher sense of embodiment through a more immersive experience and allowing for the simulation of high fidelity in a safe manner [76,77,78,79]. However, specific feasibility tests must be run, since an excessive mismatch between real and virtual movements with an HMD might cause cyber-sickness effects [80,81] and serious losses of balance [82]. Ultimately, another important challenge that might be addressed concerns the possibility of using artificial intelligence to automatically adapt the game difficulty to the specific capabilities of the subject by real-time analysis of the variables and metrics recorded by the system [83].

## Data Availability

The data presented in this study are available on request from the corresponding author.
